# Study of expansion of porcine bone marrow mesenchymal stem cells on microcarriers using various operating conditions

**DOI:** 10.1186/1753-6561-5-S8-P100

**Published:** 2011-11-22

**Authors:** Caroline Ferrari, Frédérique Balandras, Emmanuel Guedon, Eric Olmos, Nguyen Tran, Isabelle Chevalot, Annie Marc

**Affiliations:** 1 Laboratoire Réactions et Génie des Procédés, UPR-CNRS 3349, Nancy-Université, Vandœuvre-lès-Nancy, France; 2 École de Chirurgie, Faculté de Médecine, Vandœuvre-lès-Nancy, France

## Background

Bone marrow mesenchymal stem cells (BM-MSCs) represent promising source for tissue engineering and cell therapy, due to their multipotency, immunoregulation and self-renewal properties [1,2]. The expansion phase of these cells prior to differentiation and/or injection to the patient remains a critical step. BM-MSCs are classically expanded in small scale culture systems, with low control of culture conditions. However, tissue engineering and cell therapy require very large quantities of cells that cannot be easily achieved using processes in static flasks [3]. Microcarriers, classically used for industrial large scale culture of continuous cell lines, could be advantageously applied to stem cells expansion such as BM-MSCs [4-6]. Our objective was to study the influence of some operating parameters (agitation rate, microcarrier feed) on expansion and organization (adhesion, aggregation) of porcine BM-MSCs cultivated on collagen-free microcarriers, in order to improve cell expansion while maintaining their multipotency.

## Materials and methods

BM-MSCs were extracted from the iliac crest of bone marrow of three month old pigs, and purified by adherence and self-renewal properties. Cells were expanded in T-flask and on 1.2 g/L Cytodex 1 carriers (GEHealthcare) in spinner flasks. Initial cell density was 6000 cell/cm^2^ and 30 000 cell/mL (total surface of 1000 cm^2^ and medium volume of 200 mL). Culture medium was a modified α-minimal essential medium (α-MEM, Sigma) supplemented with 10 % fetal bovine serum (FBS). All cultures were performed inside an incubator (37 °C; 5 % CO_2_). Medium was exchanged (50 % volume) every two days starting from day three. Cell nuclei were counted by flow cytometry (Guava Easycyte) after cell lysis by citric acid. Cell adhesion and aggregation were observed by optical microscopy (x 40) after methylene blue staining. Cell multipotency was assayed by using differentiation kits (Invitrogen).

## Results

**Figure 1:**
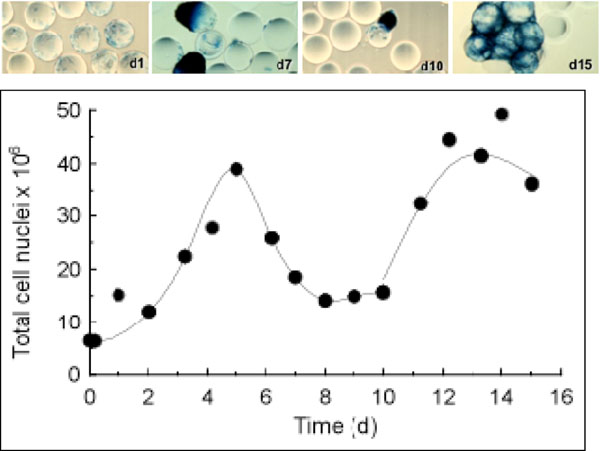
Porcine BM-MSCs growth on 1000 cm^2^ curface of Cytodex 1 stirred at 25 rpm. Addition of fresh carriers at day 10 ; 50 % medium change every 2 days

Prior to study the cell expansion phase, the adequate operating conditions of the seeding phase were determined such as the cell to bead ratio and the medium composition. Then, kinetics of cell expansion was compared between T-flask and spinner flask with cells on mirocarriers. After 8 days, BM-MSCs reached the same maximal total cell nuclei in both culture systems until cells remained as monolayer in T-flask but aggregated on microcarriers. As a consequence, cell density seemed to decrease on microccariers due to cell aggregation (Figure 1).

In a second part, growth kinetic studies of porcine BM-MSCs attached on Cytodex 1 carriers were performed at different agitation rates (0, 25, 75 rpm) in spinner flasks. Under stirred conditions, BM-MSCs cell population reached a maximal cell concentration (1.5 x 10^5^ cell/mL; x 5 multiplication factor) before to decline whatever the agitation rate used. Small aggregates was observed on microcarrier surface in the early stage of the cultures. Once those aggregates reached a critical size, they left from the microcarriers and remained in suspension, where no more growth could be observed. However, culture without agitation reached a similar maximal cell density but a longer steady phase was observed during 300 hours until cell/microcarrier clusters occurred at day 13. As cell/cell aggregation was not observed at 0 rpm, it could be assumed that Cytodex 1 surface was not directly involved in the cell aggregation phenomenon, which seemed promoted under agitation.

To verify if the aggregates were able to dissociate when exposed to new surfaces, cells were cultivated on agarose in static mode to favor aggregates. Those aggregates dissociated once exposed to static surfaces such as T-flasks or fresh Cytodex 1 carriers. Based on these observations and to allow homogeneous and controlled stirred cultures, the addition of fresh carriers during stirred cultures was further evaluated.

Firstly, fresh microcarriers were added in stirred spinner flask at day 10, when cell aggregates were already formed and cell density decreasing. Cells were able to colonize the new added surface, and to grow again, showing that cell aggregation can be reversed by adding fresh carriers. In a second time, fresh carriers were sequentially added at day 4, 8 and 12. As a result, after 12 days, total cell nuclei reached values 3 times higher than in the culture without carrier addition, suggesting that cell aggregation could be prevented by early addition of fresh carrier in the stirred culture.

Following expansion in T-flask or on microcarriers, BM-MSCs were harvested by trypsination and tested for their multipotency. Their similar ability to differentiate in adipocytes, chondrocytes and osteocytes indicated that the multipotency was preserved after cell expansion on Cytodex 1 carriers.

## Conclusion

BM-MSCs culture on Cytodex-1 collagen-free carriers in stirred systems allowed the cell expansion while maintaining their multipotency. By adding fresh microcarriers, cell aggregation could be prevented and the expansion phase duration extended. This culture process is expected to be transferred to a larger controlled culture system, in order to fulfill the need of high quantities of multipotent BM-MSCs.
